# Phenological responses to temperature are similar across spatial and temporal gradients - lessons learned from European long-term data

**DOI:** 10.1007/s00484-026-03308-z

**Published:** 2026-10-07

**Authors:** Andrea L. Silva-Cala, Robert Rauschkolb, Solveig Franziska Bucher, Jens Kattge, Sönke Zaehle, Christine Römermann

**Affiliations:** 1 Plant Biodiversity Group, Institute of Biodiversity, Ecology and Evolution, Friedrich-Schiller University Jena, Jena, Germany; 2 German Center for Integrative Biodiversity Research (iDiv) Halle-Jena-Leipzig, Leipzig, Germany; 3 Senckenberg Institute for Plant Form and Function (SIP), Jena, Germany; 4 Max Planck Institute for Biogeochemistry, Jena, Germany; 5 International Max Planck Research School (IMPRS) for Global Biogeochemical Cycles, Jena, Germany

**Keywords:** Space-for-time substitution, Flowering phenology, Space-time equivalence, Herbaceous species, Climate change

## Abstract

Understanding plant phenology patterns under global warming is important because they influence many processes, from specific dynamics, such as species interactions, to ecosystem functions, for instance, carbon uptake. Studying phenological changes requires long-term data covering the species’ distribution. However, such data is typically only locally available and restricted to certain phenophases, e.g., flowering. One common alternative is to use the space-for-time substitution (SFTS) approach. However, this method relies on the rarely tested space-time equivalence assumption. In this study, we assessed the space-time equivalence of flowering phenology by comparing its response to temperature along temporal and spatial gradients. Furthermore, we evaluated the influence of recent climate change on this assumption, analyzing the responses between 1950-1985 and 1986–2022. We used mean annual temperature data from the Era5-Land product and long-term flowering onset records of 23 herbaceous and dwarf shrub species from the PEP725 database. We found that flowering responses to temperature were mostly negative, and the responses were 67% similar between both gradients. However, the response variation along the spatial gradient did not represent the total variation observed in the temporal one. The similarity in responses between both gradients was higher (73.6%) in warmer decades (73.6% in 1986–2022 compared to 65.8% in 1950–1985), suggesting that high temperatures may synchronize phenology across space and time. We conclude that the SFTS predicts the phenological response direction to the temperature correctly. However, we recommend considering that the use of spatial gradients may not reveal the full expected response variation to temperature in a temporal gradient, especially when making general conclusions from species-specific inferences.

**Supplementary Information:**

The online version contains supplementary material available at https://doi.org/10.1007/s00484-026-03308-z.

## Introduction

Plant phenology is sensitive to climate conditions, and its changes can be interpreted as an indicator of the impacts of climate change on terrestrial ecosystems (Bandoc et al. 2022; Richardson et al. 2013; Suonan et al. 2019). In boreal and temperate ecosystems, phenology is mainly driven by temperature, number of daylight hours (photoperiod), winter chilling days, species characteristics, and individual genetic variation (Baumgarten et al. 2021; Körner and Basler 2010; Menzel et al. 2020; Rauschkolb et al. 2024; Richardson et al. 2013; Wang et al. 2020). Over the past decades, several studies have provided evidence on the impact of higher temperatures on plant phenology. In particular, changes have been observed in spring phenology, with an advance of leaf emergence and the first flowering day (Chu et al. 2023; Crimmins et al. 2010; Huang et al. 2019; Lee et al. 2022; Miller-Rushing and Primack 2008). Different species, growth forms and habitats, including polar tundra habitats and dry habitats have shown this pattern (König et al. 2018). Although most studies on plant phenology have focused on tree species, non-woody species have been gaining attention in phenological research (Deilmann et al. 2024; Diekmann 1996; Nordt et al. 2021; Rauschkolb et al. 2024). Especially because non-woody species account for a large percentage of biodiversity in temperate ecosystems, having a significant ecological impact and providing essential ecosystem services (Ellenberg et al. 2010; Gilliam 2007).

Global temperature has been increasing at an unusual rate, making the last decades the warmest in centuries (IPCC 2021). This warming has left its mark on plant phenology. For example, analysis of the five longest phenological records, including cherry blossom in Japan and tree leaf emergence in the United Kingdom, shows that these phenological events began to occur earlier from the mid-20th century (Vitasse et al. 2022). Specifically, they observed a marked advance in the mid-1980s, coinciding with the rise in temperature caused by climate change. Büntgen et al. (2022) also found an association between earlier onset of flowering with an increase of approximately 1℃ in temperature in the mid-1980s in the UK. Similarly, other studies also show a link between the advancement of such phenological events and the acceleration of warming after the middle of the 1980 s (Calinger and Curtis 2023; Calinger et al. 2013; Menzel 2000; Schaber and Badeck 2005; Steffen et al. 2015). The phenological response to temperature is frequently estimated as the shift in the number of days of an event per 1 ℃ increase in temperature. According to previous research, flowering phenology of both woody and herbaceous species has advanced by approximately 2.5 to 5 days for every ℃ (Iwanycki Ahlstrand et al. 2022; Menzel et al. 2006; Vogel 2022; Willems et al. 2022; Wittich and Liedtke 2015; Wolkovich et al. 2012). Knowing and understanding plant phenology changes caused by increased temperatures is crucial, as phenology has a strong influence on population dynamics, species composition, and species interactions (Camargo et al. 2023; Pelayo et al. 2021; Primack et al. 2009). Additionally, plant phenology drives critical ecosystem functions such as carbon uptake, photosynthetic activity, and nutrient cycling (Cleland et al. 2007; Piao et al. 2007).

Phenological data can be obtained using diverse methodologies, including human observation, herbarium specimens, near/surface digital cameras (PhenoCams), and remote sensing data (Katal et al. 2022; Tang et al. 2016). Long-term monitoring of plant phenology based on human observation offers valuable data that provides insight into phenological patterns. It additionally enables the evaluation of climate change impacts on phenology at the individual and ecosystem levels (Elmendorf et al. 2016). However, maintaining a phenological monitoring system requires high cost and effort (source and time-consuming), since ideally, monitoring is expected to be carried out regularly over a long period of time and cover the species’ distribution. There are some coordinated phenology networks providing long-term data, such as the Pan European Phenological Network (PEP), the US National Phenological Network (NPN), and the Chinese Phenological Observation Network (CPON). However, these monitoring programs face the challenges of little funding and/or difficulties finding long-term volunteers to conduct phenological observations consistently (Doi et al. 2021; Yuan et al. 2021). New technologies have given rise to other sources of phenological data, such as pictures captured with mobile phones for plant identification in an app (e.g., Flora Incognita, iNaturalist) but the phenological information available only covers the last decade (Dinnage et al. 2024; Katal et al. 2023; Klinger et al. 2023).

The space-for-time substitution method (SFTS) is frequently employed as an alternative to time series data to understand and predict the relationship between biotic variables and climatic variables (Blois et al. 2013; Bucher et al. 2019; Körner 2007; Lovell et al. 2023). For example, results obtained from elevational gradients were used to make inferences over time (Bucher and Römermann 2020). Typically, the spatial environmental gradient is based on elevation, latitude, or urbanization gradients along which temperature and other environmental factors differ substantially (Bucher et al. 2018; Buyantuyev et al. 2012; De Frenne et al. 2013; Gao et al. 2020; Wohlfahrt et al. 2019). Thus, the SFTS method appears to represent a good alternative to the lack of high-quality phenological data time series, which are often locally limited and restricted to specific world regions (Buyantuyev et al. 2012). It also provides the opportunity to integrate data from citizen science projects, as these observations can cover a broad spatial distribution with minimal effort (Crimmins and Crimmins 2022; Iwanycki Ahlstrand et al. 2022; Katal et al. 2023; Maruyama et al. 2022). However, the applicability of the space-for-time substitution approach for making inferences about changes in phenology and their consequences remains mainly unevaluated (but see Auestad et al. 2023; Blois et al. 2013; Jochner et al. 2013; Kharouba and Williams 2024).

**Fig. 1:**
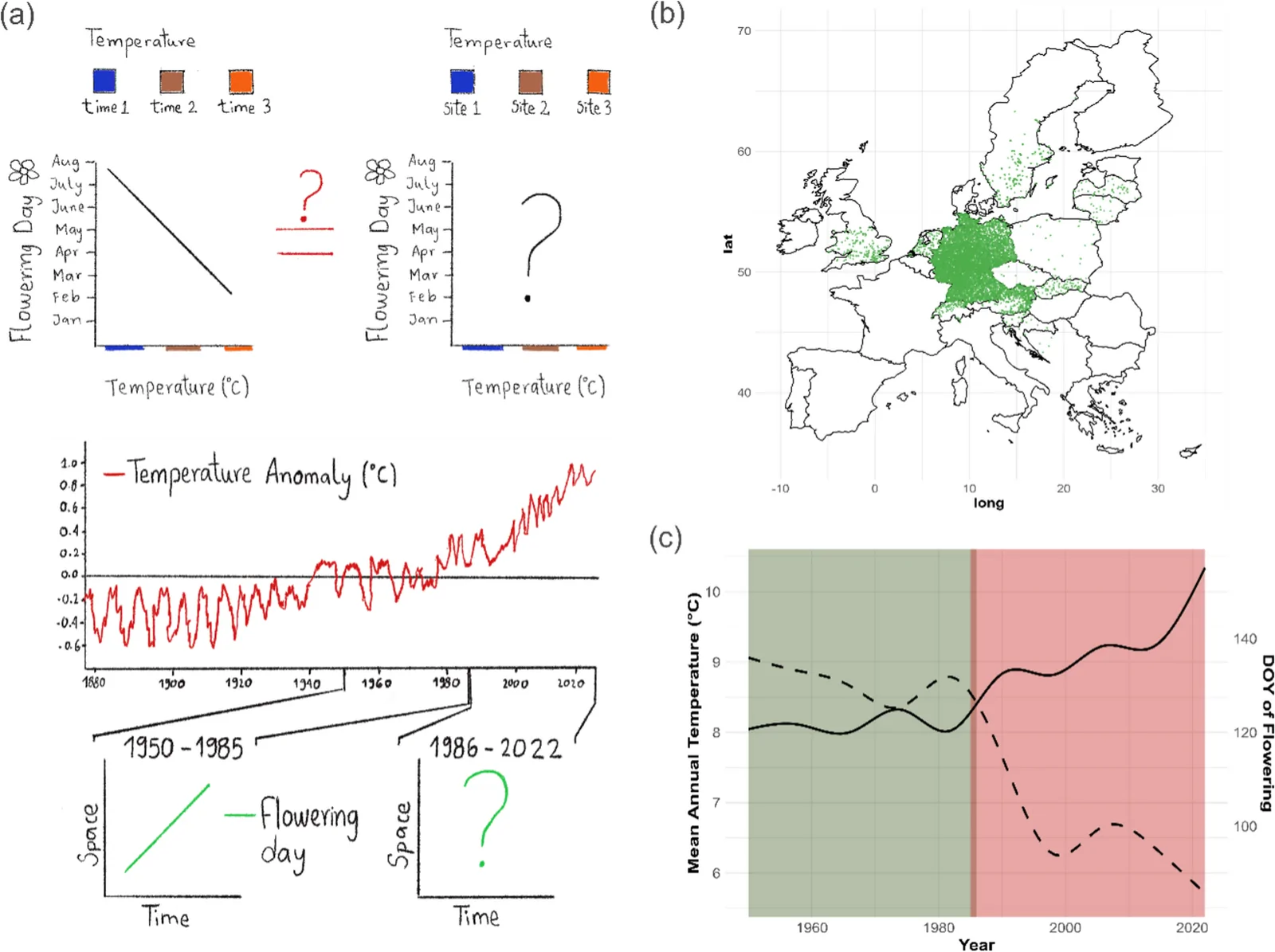
(**a**) Conceptual diagram of the hypotheses about the space-time equivalence in flowering phenology (top), and of the influence of higher temperatures in this equivalence (bottom). The graph of the temperature anomaly was made based on the time series of annual global surface temperature anomaly by NASA GISS. (**b**) Monitored stations. Each point represents a phenological monitoring site included in the study. In total, there are 7341 sites distributed across Northern, Western, and Central Europe. (**c**) Relationship between the rising temperature and the advances of flowering phenology. Mean annual temperature (solid line) and average day of the year (DOY) of first flowers (dashed line) across the sites in the periods of 1950 to 1985 (green background), where the temperature increased and phenology advanced with low intensity, and 1986 to 2022 (red background), where the temperature increase accelerated and flowering phenology occurred early in the growing season as a response. Data from the periods 1950–1985 and 1986–2022 was used (see Table 1)

The SFTS method assumes a space-time equivalence, which means that the biotic-abiotic relationship observed in space reflects the variable’s relationship in time. Although this assumption is crucial in validating the use of SFTS, it has rarely been tested and demonstrated (Lovell et al. 2023). Knapp et al. 2025 conducted a recent study that concluded that this assumption is generally applicable to herbaceous species, though their study is limited to botanical gardens, where environmental conditions, such as irrigation, are regulated. In this study, we aimed to determine whether the SFTS approach is suitable for drawing conclusions about phenological changes over time from spatial data. Specifically, we evaluated the space-time equivalence assumption using flowering phenology observations of 23 perennial herbaceous and dwarf shrub species, from the Pan European Phenology database (PEP275). By comparing the phenological response to temperature along the spatial gradient with the response along the temporal gradient, we aimed to answer the following questions (Fig. 1a):

1. Is the space-time equivalence assumption fulfilled for flowering phenology or more specifically, is the pattern of phenological response to temperature similar between the spatial gradient and the temporal gradient?
2. Do all species show the same response pattern, or are temperature responses of phenology across time and space species-specific?
3. Has the rise in temperature caused by climate change, especially since the mid-1980s, affected the space-time equivalence in flowering phenology? And does it impact all species equally?

## Methodology

### Phenological data

We obtained long-term phenology observations of the onset of flowering from the Pan-European database (PEP, http://www.pep725.eu/) (Templ et al. 2018), a weekly ground-based phenology monitoring network. Observations have been carried out by volunteers at permanent sites, who monitor various phenological events, noting the date and day of the year (DOY) when the event is observed (Templ et al. 2018). In September 2023, we accessed the flowering phenology data of 42 perennial herbaceous plant and dwarf shrub species observed between 1950 and 2023. We excluded observations from 2023 because at the time we accessed the data, the database was not yet complete for that year. We also removed any species monitored at fewer than four sites and years, as well as any sites with less than four years of data. In this way, we guaranteed that the models could be fit by species and sites in subsequent analysis. After data selection, our dataset for subsequent analysis consisted of 19 herbaceous species and 4 dwarf shrub species (Table S1), with observations between 1950 and 2022 at 7341 sites across central, north, and Western Europe (Fig. 1b).

In the process of data selection, we defined the phenological stage of flowering onset (DOY_Flowering_) by merging the phenological phases *Beginning of flowering: first flowers open* and *Beginning of flowering: 10% of flowers open*. This was done because for some locations only the second phase was monitored. The mean DOY of the two phenophases has no large difference, and the standard deviation shows that the two phases overlap (Table S2).

Based on the data obtained, we prepared different datasets for analysis according to the research questions. We divided the data into the Spatial Gradient (S.G) dataset and the Temporal Gradient (T.G) dataset. To evaluate the space-time equivalence assumption of SFTS and the general response of the flowering onset to temperature (Fig. 1a, top), we grouped the data by year to analyze the flowering phenology across different sites within each year for the S.G dataset. For the T.G dataset, we grouped the data by site to analyze the flowering phenology at each site across different years.

To disentangle species-specific patterns, for S.G, we combined species and year (sp_year), and for T.G, we combined species and sites (sp_site) (see *Analysis*).

To assess whether the relationship between flowering phenology and temperature along the two gradients has changed throughout the last century due to the ongoing warming climate (Fig. 1a, bottom), we selected only those species that were monitored continuously from 1950 to 2022. We then separated the S.G and T.G datasets in the periods 1950 to 1985 and 1986 to 2022 to differentiate between the period with nearly stable temperatures and the period of continuous acceleration of global warming, respectively (Fig. 1c). These periods were also used to show phenological changes due to climate change in Büntgen et al. (2022) and Vitasse et al. (2022). An overview of the datasets can be seen in Table 1.

**Table 1:** Overview of the data

Dataset	*N*° Sites	*N*° Years	*N*° Obs
S.G 1950–2022	7341	73	727,511
T.G 1950–2022	7205	73	717,796
S.G 1950–1985	6197	36	441,119
S.G 1986–2022	4543	37	232,758
T.G 1950–1985	5983	36	432,852
T.G 1986–2022	4019	37	225,797

Ground-based observations of the onset of flowering of 23 herbaceous and dwarf shrub species between 1950 and 2022. The datasets in the spatial gradient (S.G) and in the temporal gradient (T.G) used in the analyses are shown. Number of sites (N° Sites), number of years (N° Years), and observations (N° Obs). The observation number is the number of records for the flowering onset per species, site, and year as extracted from the Pan-European database (PEP, http://www.pep725.eu/)

### Temperature data

We accessed monthly air temperature data at 0.1° spatial resolution from the Era5-Land product (Copernicus Climate Change Service, 2019) and calculated the annual mean temperature (T_Annual_). We employed Tukey’s method (Tukey 1977) to establish the same temperature range for the S.G and T.G datasets in order to guarantee that they had the same temperature gradient and, so that the phenological response was comparable. Using this approach, the annual temperature range was established as 5 °C to 12 °C for the study sites and years (5℃ ≥ T_Annual_ ≤ 12℃). Then, we scaled and centered the temperature for subsequent analyses using the scale function in R (Becker et al. 2018). Although the annual mean temperature does not necessarily correspond to the thermal conditions required for the flower onset, it can serve as a proxy for spring temperatures, as changes in the annual average temperature correspond to changes in spring temperatures (Luterbacher et al. 2004; Twardosz et al. 2021).

### Analysis

To evaluate whether the SFTS fulfills the space-time equivalence assumption for the flowering phenology, we estimated the response of flowering time to temperature in a spatial and temporal gradient. For this, we implemented linear mixed-effect models fitted with restricted maximum likelihood using the R lmer function from lme4 package (Bates et al. 2015). In the models, DOY_Flowering_ was the response variable, and T_Annual_ was the explanatory variable. We selected the best model based on the Akaike Information Criterion (AIC) used in the step function of the lmerTest R package (Kuznetsova et al. 2017).

We fitted the models with a fixed intercept and random slopes. In preliminary analyses (not shown in this study), we executed the model with random intercepts and random slopes, however, the model failed to converge. Consequently, we used a fixed intercept. We tested two types of models: (1) to estimate the general response patterns to temperature and (2) to estimate the species-specific response patterns to temperature. For each model type, we executed a model for the spatial and temporal gradients independently. The main difference in the models' formulas was the grouping variable in the random effect term. For model (1), in the S.G, we assigned the years as the grouping variable in the random effect, so the model estimated a regression slope (the phenological response) between DOY_Flowering_ and T_Annual_ at different sites (spatial gradient) for each year. In the case of T.G, we assigned sites as a random effect, estimating a regression slope for each site over the years (temporal gradient).

To include the species in the grouping variable for model (2), we joined the species name to the years and sites, resulting in the creation of the new variables sp_year and sp_site. In the S.G, we assigned the sp_year variable in the random effect term, and in the T.G, we assigned the sp_site variable in the random effect. In this way, the models’ outputs included a regression slope between different sites for each species-year in the S.G and between different years for each species-site in the T.G.

1. Model to evaluate general patterns:

For the spatial gradient (S.G dataset):

$$1mer\;\begin{pmatrix}DOY_{Flowering}\sim T_{Annual}\\+\left(0+T_{Annual}\vert year\right)\end{pmatrix}$$

Where,

*DOY*_*Flowering*_ is the dependent variable and represents the day of the year (DOY) that flowering onset occurred.

*T*_*Annual*_ is the fixed effect and represents the annual temperature. This makes it possible to assess the effect of temperature on the flower onset date.

*(0 + T*_*Annual*_*| year)* is the random effect.

*year* is the grouping variable. It means that the data is nested within years. It also determines the spatial gradient, as for each year, the temperature variation comes from the different geographical sites. For example, for the year 2010, the slope estimated in the model represents how much the flowering onset shifted per temperature unit across the spatial gradient in that specific year.

*0 + T*_*Annual*_ represents the intercept and slope, respectively. *0* means the intercept is fixed and does not change. *+T*_*Annual*_ means random slopes. It allows calculating a response to temperature (slope) for each *i* in the grouping variable.

For the temporal gradient (T.G dataset):

$$1mer\;\begin{pmatrix}DOY_{Flowering}\sim T_{Annual}\\+\left(0+T_{Annual}\vert site\right)\end{pmatrix}$$

Where, in contrast to the previous model,

Site is the grouping variable, meaning the data is nested within sites. It also determines the temporal gradient, as for each site, the temperature variation comes from the different years (time). For example, for the site “A”, the slope estimated in the model represents how much the flowering onset shifted per temperature unit over the temporal gradient in that specific site.

We extracted the slopes obtained from the models of both gradients and compared them using two analyses. First, we implemented a t-test (Student 1908) to compare the mean slopes of the S.G and T.G and evaluated whether the mean response to temperature is significantly similar between the two gradients. Additionally, we compared the distribution of the slopes using the Jensen-Shannon distance (JS). The JS distance is based on the Kullback-Leibler (KL) divergence (Kullback and Leibler 1951) and quantifies the similarity between two probability distributions, considering the asymmetry, shape, and dispersion of the distributions. This metric returns a distance or similarity value ranging from 0 to 1, where values closer to zero indicate greater similarity. (Lin 1991; Nielsen 2019).

(2) Model to evaluate species-specific patterns:

For the S.G:

$$1mer\;\begin{pmatrix}DOY_{Flowering}\sim T_{Annual}\\+\left(0+T_{Annual}\vert sp\_year\right)\end{pmatrix}$$

Where,

*sp_year* is the grouping variable. We created this variable by joining the species name and the years. This variable determines the spatial gradient (see (1) Model). The inclusion of the species name enables the model to estimate a slope for each species-year combination, thereby accounting for the species-specific response to temperature.

For the T.G:

$$1mer\;\begin{pmatrix}DOY_{Flowering}\sim T_{Annual}\\+\left(0+T_{Annual}\vert sp\_site\right)\end{pmatrix}$$

Where,

*sp_site* is the grouping variable. We created this variable by joining the species name and the sites. This variable determines the temporal gradient. The inclusion of the species name enables the model to estimate a slope for each species-site combination, thereby accounting for the species-specific response to temperature.

To test the impact of climate change on phenological response in S.G and T.G, we fitted the models using the same parameters as described above in (1) and (2). In this case, we also evaluated the general response pattern as well as species-specific patterns for the periods from 1950 to 1985 and from 1986 to 2022. We compared the distributions of the slopes obtained for each period between both gradients, also using JS and t-tests. In addition, we compared the DOY_Flowering_ of the two periods using a t-test.

To present the estimated slopes in days/°C, we had to divide them by the standard deviation of the unscaled and uncentered T_Annual_ data, as in our models we utilized scaled and centered annual mean temperature.

For all analyses described above, we had a large difference between the number of slopes estimated in the models for the S.G. and the T.G. (the numbers of slopes in the T.G. were always greater than in the S.G.). So, we generated 1000 samples of the model slopes from the T.G dataset. Each sample size was made equivalent to the number of slopes in the S.G dataset. For models designed to assess species-specific patterns, we generated the samples per species, ensuring each sample contained slopes from all species. We then calculated the JS distance between each of these samples and the slopes from the T.G dataset. Finally, we compared the JS distance obtained from all the samples with the JS distance from the original data using either a t-test or Wilcoxon test (Wilcoxon 1945), depending on the distribution (Table S2-S4).

## Results

### Space-time equivalence assumption in flowering phenology

In general, the model results suggested that temperature is a substantial driver of the flowering onset. Specifically, our models estimated that the start of flowering has a sensitivity of −7.08 days/°C in the S.G and − 8.95 days/°C in the T.G (Table 2). However, when we looked at the slopes of the random effects, we observed that in the T.G, 6.5% of the slopes (470 of 7205) were positive, meaning that in these cases the phenology is delayed as temperature rises, while in the S.G, all the slopes (73) we obtained were negative.

**Table 2:** Results of the linear mixed-effects models comparing the response of the flowering onset to temperature across the spatial and temporal gradient

Spatial Gradient					Temporal Gradient				
Random Effects	Obs	Variance	SD	p-value	Random Effects	Obs	Variance	SD	p-value
Years	73	10.91	3.304	**< 0.001**	Sites	7205	102.98	10.15	**< 0.001**
Residual		4707.51	68.61		Residual		4618.41	67.96	
Fixed Effects		*Estimates*	*SE*	*p-value*	*Fixed Effects*		*Estimates*	*SE*	*p-value*
Intercept		123.6	0.10	**< 0.001**	Intercept		124.02	0.10	**< 0.001**
T_Annual_		−8.57	0.40	**< 0.001**	T_Annual_		−10.82	0.17	**< 0.001**
Marginal *R*^2^	0.015	*Conditional R* ^2^		0.018	*Marginal R* ^2^	0.024	*Conditional R* ^2^		0.045

Variance of the random effects and estimates of the fixed effects for both gradients are shown. *SD* Standard deviation, *SE* standard error. Estimates of T_Annual_ are in days/SD of °C. Models formula: lmer (DOY_Flowering_ ~ T_Annual_ + (0 + T_Annual_ | year)) for the spatial gradient, lmer (DOY_Flowering_ ~ T_Annual_ + (0 + T_Annual_ | site)) for the temporal gradient

**Fig. 2:**
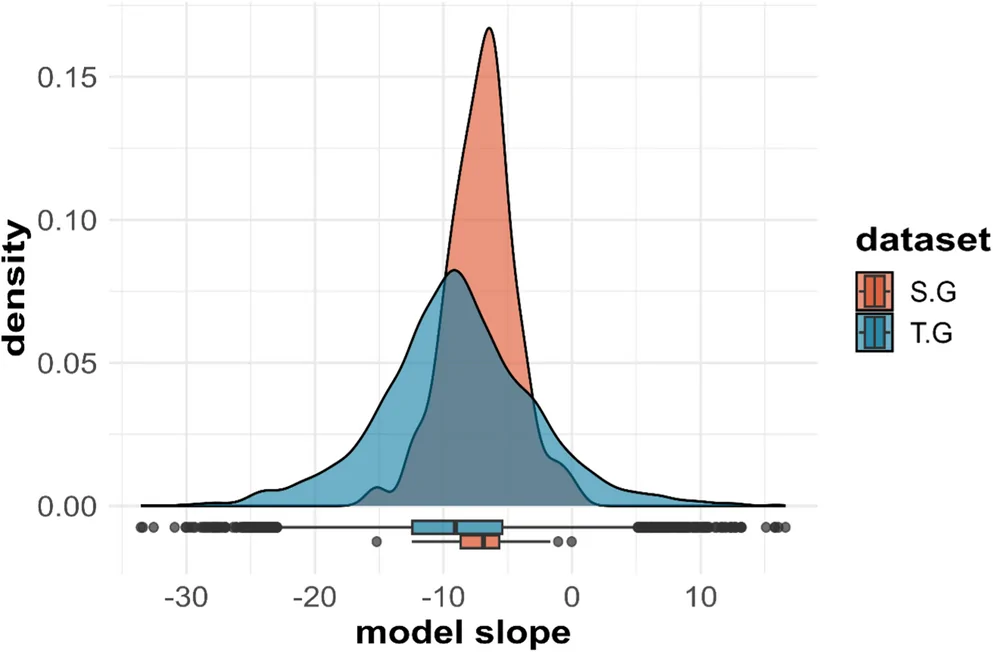
Distribution of the phenological response to temperature across the spatial (S.G in red) and temporal gradient (T.G in blue) as a density plot and boxplot. The phenological response has greater variation in T.G, while in S.G, the response is more homogeneous. The Jensen-Shannon (JS) distance between both distributions is 0.33, indicating that they are 67% similar

When we compared the models’ slope distribution, the JS distance indicated that the phenological response exhibits 67% similarity across both temporal and spatial gradients (JS = 0.328). The slope distribution in the T.G showed higher variation than in the S.G, where the slope distribution was more homogeneous (Fig. 2). In the T.G, the slopes ranged from ₋33.6 to + 16.6, and in the S.G the slopes ranged from − 15.2 to −0.02. The slope means across the two gradients were significantly different to one another (*t* = 5.84; p-value < 0.001).

### Species-specific responses

**Fig. 3:**
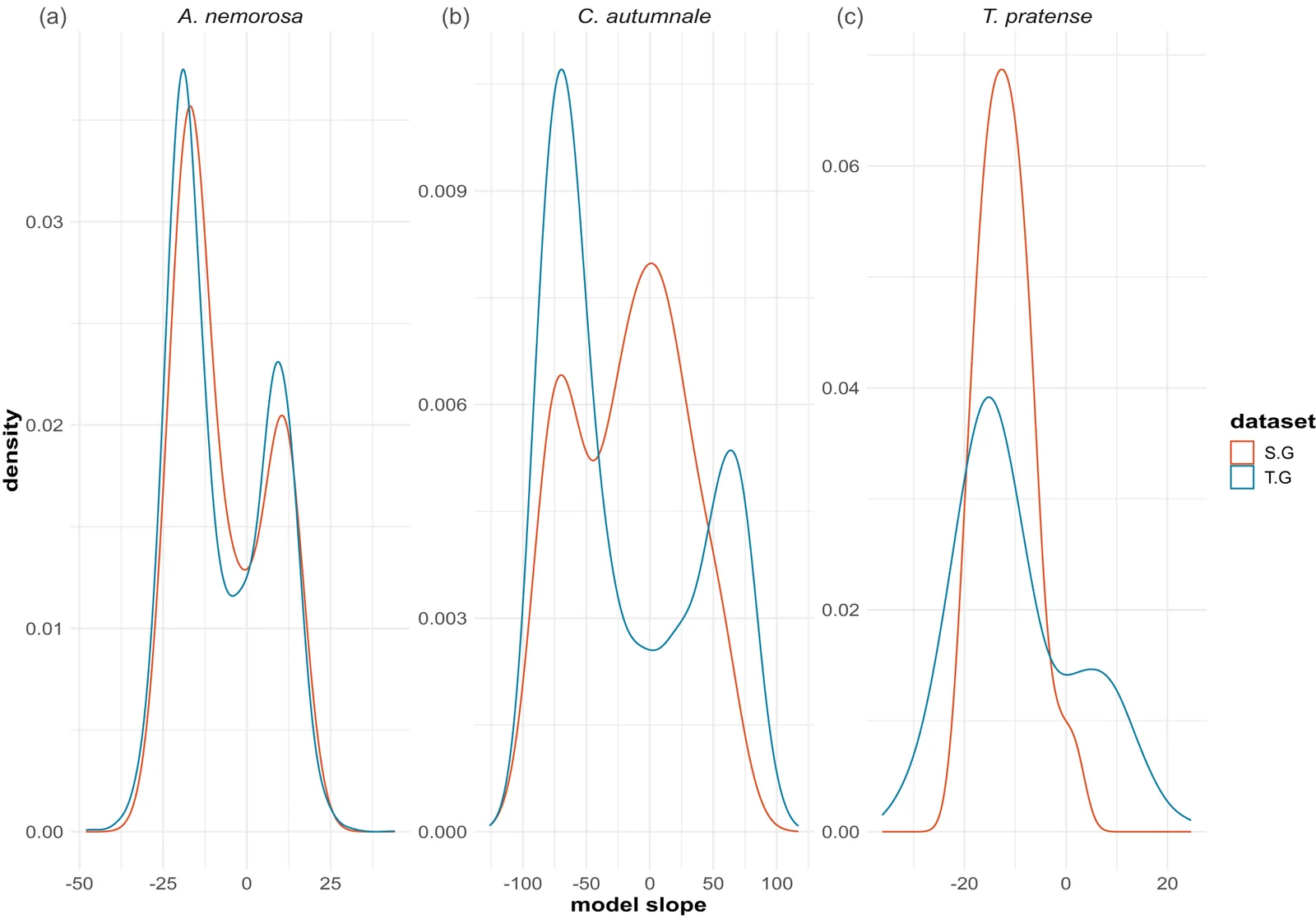
Density plots of model slopes showing the three primary patterns of phenological response to temperature observed: (**a**) similar, (**b**) divergent, and (**c**) varying magnitudes between spatial (S.G, red) and temporal (T.G, blue) gradients. Each panel displays the response for a single representative species, *Anemone nemorosa* in (**a**), *Colchicum autumnale* in **(b**), and *Trifolium pratense* in (**c**)

The models testing for the species effect show that each species has their own response pattern, that is, the spatial-temporal patterns of phenological response are species-specific (Figure S1). Some species showed very similar responses in both gradients, while others showed slightly divergent responses, and others showed similar responses but with differences in the magnitude between both gradients (Fig. 3). Specifically, species such as *Anemona nemorosa*, *Calluna vulgaris*, *Galanthus nivalis*,* Leucanthemum vulgare*,* Rubus idaeus*,* Tussilago farfara*, and V*accinium myrtillus* showed high similarity (> 87%) in their phenological response to temperature between both gradients. Conversely, species like *Leucojum vernum*,* Dactylis glomerata*, and *Viola odorata* showed the lowest similarity (< 62%) between S.G and T.G. Still other species, including *Trifolium pratense*, *Fragraria vesca*, and *Primula veris*, exhibit differences in the magnitude of their responses. The specific Jensen-Shannon distance for each species as well as the mean response (mean of the slopes) across both gradients is detailed in Table 3. Overall, the slopes vary considerably when the species are incorporated into the random effect in the model (Figure S2, Table S5). More than 30% of the flowering onset-temperature relationships (DOY_Flowering_ ~ T_Annual_) were positive. Though overall, the negative relationship was maintained with a sensitivity of −5.16 days/°C in the S.G and − 7.6 days/°C in the T.G (Table S6).

**Table 3:** List of species with the Jensen-Shannon (JS) distance and the mean response to temperature across the spatial and temporal gradient

Species	JS distance	Similarity (%)	Mean Slope S.G	Mean Slope T.G
*Eranthis hyemalis*	0.209	79	−4.66	−23.36
*Galanthus nivalis*	0.112	89	−14.60	−13.40
*Leucojum vernum*	0.396	60	9.29	3.32
*Anemone hepatica*	0.390	61	9.64	1.19
*Tussilago farfara*	0.103	90	−8.27	−8.61
*Viola odorata*	0.433	57	5.07	−1.46
*Anemone nemorosa*	0.071	93	−6.88	−7.61
*Primula veris*	0.241	76	−2.47	−4.08
*Caltha palustris*	0.178	82	−1.63	−2.72
*Oxalis acetosella*	0.256	74	−5.36	−6.16
*Fragaria vesca*	0.255	75	−4.82	−5.55
*Convallaria majalis*	0.178	82	−6.30	−6.14
*Vaccinium myrtillus*	0.097	90	−5.72	−6.05
*Rubus idaeus*	0.101	90	−10.50	−7.82
*Dactylis glomerata*	0.389	61	−8.34	−6.07
*Trifolium pratense*	0.334	67	−11.82	−9.84
*Vaccinium vitis-idaea*	0.165	84	−11.81	−12.87
*Leucanthemum vulgare*	0.120	88	−16.73	−15.77
*Epilobium angustifolium*	0.154	85	−20.11	−20.23
*Artemisia vulgaris*	0.153	85	32.30	23.32
*Cyclamen purpurascens*	0.361	64	−26.29	−15.63
*Calluna vulgaris*	0.129	87	5.66	−2.50
*Colchicum autumnale*	0.237	76	−16.28	−20.89
**MEAN**	**0.22**	**78**	**−5.24**	**−7.34**

The species are listed in ascending order based on their flowering times. The JS distance per species and their respective percentage similarity values are shown, as well as the mean of the slopes in the spatial (S.G) and temporal gradient (T.G). The range of the JS distance is from 0 to 1, where the closer to zero, the more similar the distributions are

### Space-time equivalence during recent climate change

**Fig. 4:**
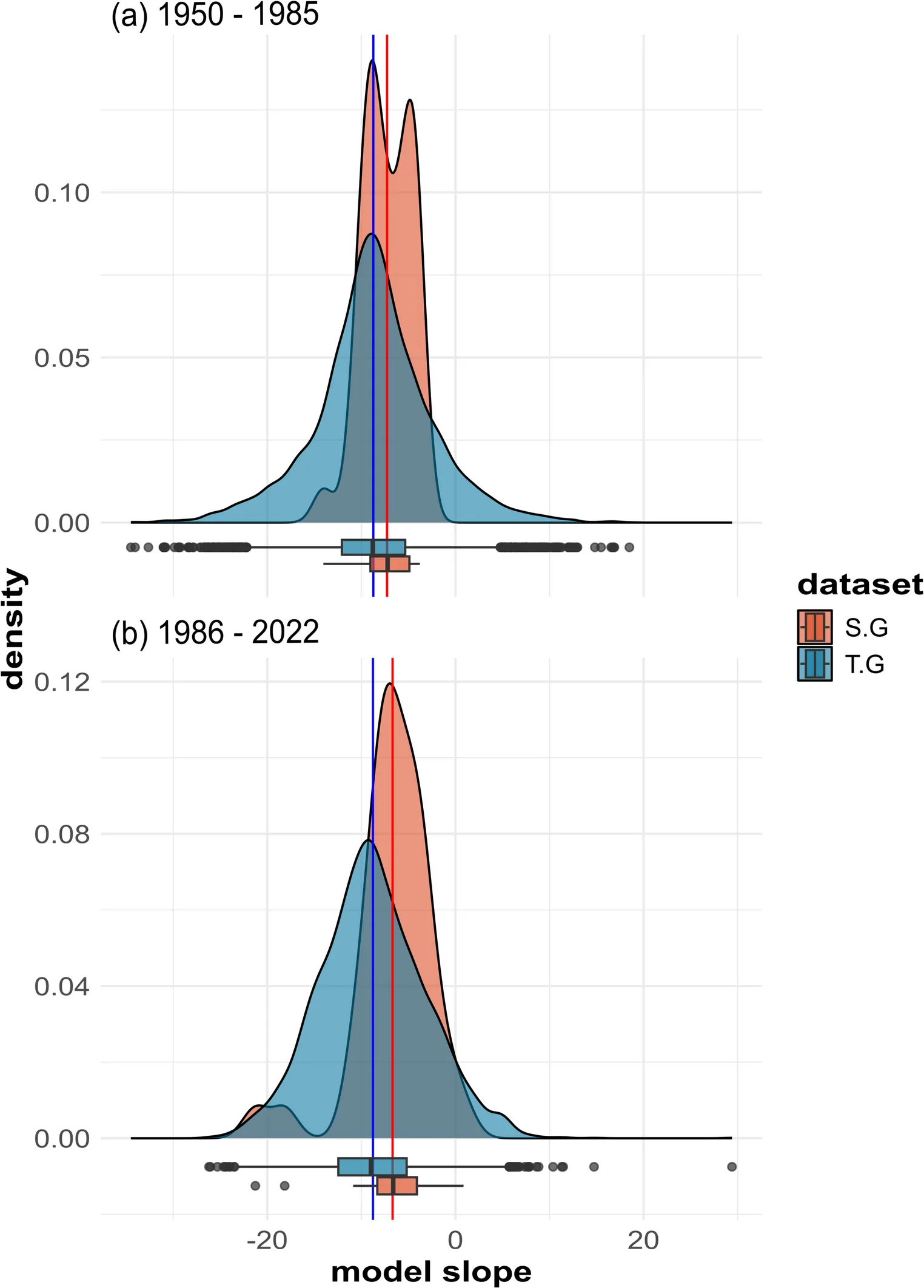
Effect of temperature on flowering phenology before and after 1985 along the spatial and temporal gradient. Density plot and boxplot of the spatial (red) and temporal (blue) gradient slopes, (**a**) in the period 1950 to 1985 and (**b**) in the period 1986 to 2022. The vertical lines show the mean value of the slopes, (**a**) S.G: −7.27, T.G: −8.73; (**b**) S.G: −6.68, T.G: −8.77

Our findings indicated that the phenological response to temperature has become more consistent across the S.G and T.G in the recent period from 1986 to 2022 (Fig. 4). The JS distance showed that the phenological response was 73.6% similar between both gradients (JS = 0.263). In the early period from 1950 to 1985, we calculated a similarity of 65.8% (JS = 0.342). Specifically, we observed that in the T.G the response was more homogeneous in the recent period, showing a narrower slope distribution (less variation), while in the S.G, the response was slightly more heterogeneous, exhibiting more negative slopes than in the earlier period (Fig. 4).

We did not observe any marked difference in the phenological sensitivity between the two periods. For the recent period, the models estimated a sensitivity of −6.7 days/°C in the S.G and − 8.7 days/°C in the T.G. In the earlier period, we obtained a sensitivity of −7.3 days/°C in the S.G and − 8.7 days/°C in the T.G (Table S8). However, the mean of DOY_flowering_ in the 1986 to 2022 period was around 26 days earlier (mean DOY_flowering_ = 104) compared to the 1950 to 1985 period (mean DOY_flowering_ = 130), being significantly different (*t* = 153.36; p-value < 0.001) (Fig. 1c). In both periods, the average phenological response (mean of the slopes) was significantly different between both gradients (t-test, 1950–1985: *t* = 4.3; p-value < 0.001; 1986–2022: *t* = 3.02; p-value < 0.01).

In comparison, in the models combining species with the sites or years as random effects, we found an increase in the temperature sensitivity of the flowering phenology in the most recent period compared with the earlier one (1950–1985 period: 4.7 days/°C and 7.6 days/°C in S.G T.G, respectively; 1986–2022 period: 8.1 days/°C and 8.8 days/°C in S.G T.G, respectively) (Table S7). We did not find a general pattern in the similarity of the phenological responses between the two periods (Table 4). However, we observed that some species seem more affected than others by higher temperatures. For example, some species showed more negative slopes in the recent period (1986–2022) (such as *Convallaria majalis*,* Colchicum autumnale* and *R. idaeus*), while others maintain a wide variation in their response in both periods and in both gradients (such as *C.* v*ulgaris*) or showed more positive slopes (such as *A. hepatica*) (Figure S3; Table 4).

**Table 4:** Overview of the Jensen-Shannon (JS) distances and the mean response to temperature between both gradients for the 1950–1985 and 1986–2022 periods

Species	1950–1985				1986–2022			
	JS distance	Similarity (%)	Mean Slope S.G	Mean Slope T.G	JS distance	Similarity (%)	Mean Slope S.G	Mean Slope T.G
*G. nivalis*	0.154	85	−10.9	−18.3	0.089	91	−13.9	−13
*A. hepatica*	0.455	55	7.6	−5	0.278	72	3.5	0.5
*T. farfara*	0.173	83	−5.1	−11.4	0.135	87	−7.6	−8.5
*A. nemorosa*	0.21	88	−4.6	−8.6	0.203	80	−5.3	−7
*C. majalis*	0.315	69	−4.3	−5.1	0.304	70	−14.7	−12.9
*R. idaeus*	0.055	95	−5.1	−4.2	0.274	73	−18.7	−10.8
*D. glomerata*	0.357	64	−7.8	−5.3	0.488	51	−11.8	−6.3
*C. vulgaris*	0.142	86	−3.7	5.4	0.128	87	14.1	0.2
*C. autumnale*	0.128	87	−8.7	−2.7	0.214	69	−19.2	−18.7
**MEAN**	**0.211**	**79**	**−4.7**	**−6.1**	**0.235**	**77**	**−8.2**	**−8.5**

The species are listed in ascending order based on their flowering times across both study periods. The distance per species, their respective percentage similarity as well as the mean of the slope are shown. The range of the JS distance is from 0 to 1, where the closer to zero, the more similar the distributions are

## Discussion

We found that the space-time equivalence of the flowering onset response to temperature was 67% similar across the spatial and temporal gradient. The models estimated that flowering onset occurs approximately 7 and 9 days earlier for every °C increase in mean annual temperature across the S.G and T.G, respectively. The response patterns were species-specific showing species for which the phenological response was very similar (93%, the highest) between both gradients, and others with divergent response (57%, the lowest). Furthermore, our results indicate that the response varied more along the spatial gradient and less along the temporal gradient during the 1986–2022 period, when temperatures were higher, compared to the 1950–1985 period (Fig. 4). This has resulted in a more consistent phenological response to the temperature between both gradients over the past 37 years. However, not all species are equally affected in their flowering phenology by higher temperatures in the last decades.

### Phenological response and space-time equivalence

Our results indicate that in the spatial gradient we can observe a similar pattern and the same direction of phenological response to temperature as observed in the temporal gradient. The percentage of negative slopes was 100% in S.G and 93% in T.G. Similar results were obtained using data from herbaria in the United States, where the phenological response of flowering to increasing temperature in space and time shows the same direction and magnitude (Ramirez-Parada et al. 2024). In the same way, when using phenological observations of herbaceous plants in botanical gardens in Europe, no significant differences were found in the average day of flowering onset between observations from different years and observations at different locations (Knapp et al. 2025). However, there are some differences in the phenological response between the two gradients. The main difference we observed is that the variation in the response was greater along the temporal gradient than along the spatial gradient. This indicates that the spatial gradient did not reflect the total response variation that we observed in the temporal gradient. While in the S.G, the slopes ranged from − 15.2 to −0.0252, the range of slope values in the T.G was from − 33.55 to 16.61. This means that, in some sites, flowering has advanced more markedly over the years, while in a few other sites the flowering phenology has been delayed over the years in response to temperature. In herbaceous species, delayed flowering can occur due to late snowmelt or drought in late summer of the previous year (Bucher et al. 2019; Piippo et al. 2009). The wide response variation we obtained can be explained by local environmental conditions that can influence phenology, such as water availability (Peñuelas et al. 2004; Piao et al. 2019), soil properties (Primack et al. 2009) or even elevation (Willems et al. 2022). Additionally, phenological variation is also influenced by functional traits, particularly in herbaceous species compared to tree species (Bucher et al. 2018; Horbach et al. 2023). In terms of the application of SFTS, our findings suggest that the spatial gradient accurately represents the overall pattern and direction of the response; however, it may not accurately capture the complete variation in the response that we observed in the temporal gradient. This can be a disadvantage when evaluating specific patterns or responses with SFTS, as the use of a spatial gradient in these cases could underestimate the variation in the phenology-temperature relationship. For instance, in species such as *Eranthis hyemalis* and *Primula veris*, the mean response in the spatial gradient is lower than in the temporal gradient (Table 3; Figure S1). Conversely, for understanding the general phenological response, this limitation could become an advantage because it reduces unnecessary noise, allowing us to capture the overall pattern and direction of the phenological response to increasing temperature (Fig. 2). Greater response variation is reported in studies that evaluate the phenology-temperature relationship using a spatial gradient (Cornelius et al. 2013; Dai et al. 2014; Gao et al. 2020; Vitasse et al. 2018); however, these studies do not incorporate analyses along the temporal gradient, making a direct comparison challenging.

### Species-specific responses to temperature

We showed that the space-time equivalence is clearly species-specific. Our results suggest that it would not be appropriate to draw general conclusions from individual species when using the SFTS method. We noticed that even close relatives such as *A. nemorosa* and *A. hepatica* show different response patterns to temperature and different similarity of response between the two gradients (93% and 61%, respectively). Studies have revealed that interspecific variation in phenological response to temperature increase is indifferent to phylogenetic signals (CaraDonna and Inouye 2015; Hassan et al. 2022). In fact, phylogenetic signals were found to be associated with flowering times but not with the temperature sensitivity of flowering phenology (Panchen et al. 2025). On the other hand, Knapp et al. (2025) suggest that space-time equivalence is more robust in early-flowering species than in late-flowering ones. Although we did not take the seasonality of flowering into account in our analyses, our results did not indicate a trend in the similarity of the phenological response across both gradients. For example, the early-flowering species *A. hepatica* and *A. nemorosa* showed 61% and 93% similarity, respectively; these values are comparable to the late-flowering species *C. purpurascens (*64%) and *Artemisia vulgaris* (85%).

In addition, we observed that phenological responses to temperature, both across space and time, exhibit considerable intraspecific variation. In fact, for all species, we obtained both negative and positive slopes, regardless of the flowering seasonality (Figure S1). This wide intraspecific variation can be explained by multiple factors, such as functional traits (Rauschkolb et al. 2024), different genotypes (Valdés et al. 2019) or site conditions (König et al. 2018). For example, leaf area and plant height were found to be relevant and associated with phenology variation in herbaceous species (König et al. 2018; Rauschkolb et al. 2024; Sporbert et al. 2022). Other studies show that populations growing in warm or dry conditions flower earlier and those growing in cold sites have higher temperature sensitivity (Pearson et al. 2021; Willems et al. 2022). Given that multiple factors can explain intraspecific phenological variation, it is important that when using the SFTS method, observations of several individuals along the spatial gradient are made to account for this factor (Lajoie and Vellend 2015). However, we also highlight that 22 of the 23 species analyzed show a similarity in response to temperature greater than 60% between both gradients, indicating again that the SFTS method reflects the general direction and response to temperature, which would be expected in the temporal gradient.

### Effects of climate change on space-time equivalence

We observed that the major impact of recent climate changes on the distribution of the phenological response is the reduction of its variation. Our results indicate that the space-time equivalence has been more consistent in recent decades, showing 8% greater similarity than in the earlier period. Specifically, in the T.G, the response has lower variation in the period from 1986 to 2022. These results suggest that climate change may synchronize phenology between space and time. This is especially relevant because spatial gradients enable us to evaluate the phenological response to temperature conditions that are either warmer or colder than those that are detected by temporal analyses (De Frenne et al. 2013). Some studies also indicate that global warming synchronizes phenology across elevations and suggest that this may change the ecological functions of mountain ecosystems (Vitasse et al. 2018; Yang et al. 2025). The study of Girardello et al. (2024) found evidence that higher temperatures in boreal and temperate biomes reduce phenological diversity in forest ecosystems. This is further evidence that, given the increase in temperature due to climate change, phenology is responding strongly to these changes. In fact, even with varying local conditions along spatial gradients and despite the influence of other environmental factors, the widespread phenological changes reported across numerous studies predominantly indicate a strong driving force from temperature increases due to climate change (Cleland et al. 2007; Menzel et al. 2006; Rauschkolb et al. 2024).

According to our results, the increase in the mean annual temperature over the nearly four decades (1986–2022) did not have an impact on the overall flowering time sensitivity compared to earlier periods (1950–1985). However, the result is different when we consider the variation caused by species in our models by adding them into the random effect. In this case, we evidenced greater sensitivity in recent decades (−8.1 days/°C and − 8.8 days/°C in the S.G and T.G, respectively) compared to earlier decades (−4.7 days/°C and − 7.5 days/°C in the S.G and T.G, respectively). This result indicates that the rise in temperature in recent decades has increased the sensitivity of flowering phenology, causing it to occur 8 days earlier for every 1 °C increase in the average annual temperature. This result reinforces the extensive evidence that phenology is sensitive to forcing temperature and has been advancing with warmer decades (Khanduri et al. 2008; Menzel et al. 2020; Parmesan and Yohe 2003; Richardson et al. 2013; Root et al. 2003). It is important to acknowledge that as phenological monitoring was conducted on a weekly basis, our estimates of the phenological response to temperature have a deviation of ± 7 days. However, this monitoring interval is commonly used and recommended (Cornelius et al. 2011; Nordt et al. 2021). Additionally, it has been found that more frequent monitoring does not affect the estimation of phenological changes in response to climate (Miller-Rushing et al. 2008).

Nevertheless, our results also suggest that the advance in flowering may not be solely due to the increase in temperature. The mean DOY_Flowering_ in the recent period occurs earlier than expected based on the DOY/°C relationship found in our model (~ −7 days/°C and − 9 days/°C for S.G and T.G, respectively). Our result of 26-day advance in the first flowering would mean that the mean annual temperature increased by 4 °C, but it increased by 0.85 °C. This means that between both periods there are stronger changes in flowering phenology than those explained by the change in temperature alone. Similar results were obtained by Büntgen et al. (2022), where they reported a 32-day difference in the average first day of flowering for herbaceous species in the UK, comparing observations from before 1986 with those after 1987. Additionally, their results showed this pattern to be more pronounced in herbaceous species than in trees and shrubs. Similarly, in the Mediterranean, the average flowering peak in understory plants was observed to occur 22 days earlier in the 2020 s compared to the 1980 s (Pareja-Bonilla et al. 2023).

The impact of the temperature increase over nearly four decades on space-time equivalence varies between species. One difference we observed is that some species maintain the variation in their response, while in others, this variation has decreased in the recent period. Greater variation in response to temperature among populations indicates greater phenological plasticity, which allows plants to cope with (or react to) changes in the environment (Knott et al. 2023; Vitasse et al. 2010). At the same time, the advancement of flowering phenology in response to rising temperatures may prolong the reproductive period and thus improve fitness (Song et al. 2022). Based on these observations, our results could reflect different strategies used by the species to ensure a successful flowering time in warmer conditions.

There are also studies that have found a link between temperature sensitivity and flowering seasonality. For example, late-flowering species are generally found to be less affected (less sensitive) by rising temperatures compared to early-flowering species (Auffret 2021; Geissler et al. 2023; Moore and Lauenroth 2017; Pareja-Bonilla et al. 2023). Although we did not consider the seasonality of the flowering, our results do not show a clear pattern between early-flowering and late-flowering species (Table 4). In the study conducted by Knapp et al. 2025; which also assessed the space-time equivalence in herbaceous plants, the authors found that the response to temperature along the temporal gradient was less pronounced (less negative) in late-flowering species. However, this relationship was not observed across the spatial gradient. Our results and those of Knapp et al. 2025 indicate that further research is necessary to gain a comprehensive understanding of the relationship between the seasonality of flowering in non-woody species and the phenological response to increasing temperature across spatial and temporal gradients. Additionally, the question remains whether a temperature measure that is more relevant to the flower onset, such as spring temperature, might be more precise in evaluating space-time equivalence. For example, the study of Landuyt et al. (2025) suggest that the prediction of the phenological sensitivity depends on the forcing time windows. Further warming, and more frequent extreme events, may alter the phenological response, no longer causing it to advance continuously (Iler et al. 2013; Zettlemoyer and Lesica 2026). This phenomenon could affect the applicability of spatial gradients; therefore, future studies should focus on unravelling the response to temperature and its constraints. For example, in trees, the number of cold days constrains the response to temperature (Baumgarten et al. 2021; Beil et al. 2021). In herbaceous species, snowmelt and drought could constrain or alter the phenological response to temperature (Jabis et al. 2020).

## Conclusion

Our results indicate that the phenological response to temperature in the spatial gradient reflects the general pattern of the responses observed over the temporal gradient in perennial herbaceous species and small shrubs in Europe. The increase in temperature caused by climate change has reduced the response variation in the temporal gradient leading to greater response consistency across both gradients. This suggests that the increase in temperature does affect space-time equivalence, making the response of the flowering onset more similar between the spatial and temporal gradient. Considering our findings, along with the utility of the SFTS approach in the context of limited historical data, we conclude that the use of SFTS is appropriate for phenological studies. We recommend, however, considering that the use of a spatial gradient may not reflect the full expected variation and being cautious when drawing generalized conclusions from species-specific inferences, as the phenological response may be species-specific. For future research, we suggest including tree species in the analysis because, compared to non-woody plants, they don’t react as strongly to rising temperatures, primarily due to insufficient winter chilling (Baumgarten et al. 2021; Beil et al. 2021; Wang et al. 2020). This could have a large effect on space-time equivalence across both gradients. Likewise, future studies are needed to better understand the relationship between the phenological response to temperature and the seasonality of flowering across the spatial and temporal gradients. One of the main criticisms of the use of SFTS is the lack of rigor in not evaluating assumptions; our study is a step in that direction, and our results may serve as a reference for future studies on plant phenology using SFTS.

## Supplementary Information

Below is the link to the electronic supplementary material.

Supplementary Material 1 (DOCX 1.21 MB)

## Data Availability

The phenological data from the PEP database used in this study are available at https://doi.org/10.5281/zenodo.21979471.

## Abbreviations

DOY_Flowering_: Day of the Year when flowering begins
JS: Jensen-Shannon Distance
SFTS: Space-for-time substitution
S.G: Spatial gradient
T_Annual_: Annual Mean Temperature
T.G: Temporal gradient
