# Supplementary material for: Phenological responses to temperature are similar across spatial and temporal gradients - lessons learned from European long-term data
Source: Int J Biometeorol. 2026 Oct 7;70(10):272. doi: 10.1007/s00484-026-03308-z (PMC13645973; doi:10.1007/s00484-026-03308-z)
Supplement: Supplementary file 1 [file 484_2026_3308_MOESM1_ESM.docx]

Evaluating the space-for-time substitution approach in phenology research - lessons learned from northern hemisphere long-term data

Andrea L. Silva-Cala^1,3,4,5*^, Robert Rauschkolb^1,2,3^, Solveig Franziska Bucher^1,2,3^, Jens Kattge^2,4^, Sönke Zaehle^2,4^ & Christine Römermann^1,2,3^

^1^ Plant Biodiversity Group, Institute of Biodiversity, Ecology and Evolution, Friedrich-Schiller University Jena, Jena, Germany.

^2^ German Center for Integrative Biodiversity Research (iDiv) Halle - Jena - Leipzig, Leipzig, Germany.

^3^ Senckenberg Institute for Plant Form and Function (SIP), Jena, Germany.

^4^ Max Planck Institute for Biogeochemistry, Jena, Germany.

^5^ International Max Planck Research School (IMPRS) for Global Biogeochemical Cycles, Jena, Germany

*Corresponding author.

E-mail address:

| Andrea L. Silva-Cala | andrea.silva.cala@uni-jena.de |
| --- | --- |
| Robert Rauschkolb | robert.rauschkolb@uni-jena.de |
| Solveig Franziska Bucher | solveig.franziska.bucher@uni-jena.de |
| Jens Kattge | jkattge@bgc-jena.mpg.de |
| Sönke Zaehle | szaehle@bgc-jena.mpg.de |
| Christine Römermann | christine.roemermann@uni-jena.de |

Materials and methods

| Species | Beginning of flowering: first flowers open | | Beginning of flowering: 10% of flowers open | |
| --- | --- | --- | --- | --- |
|  | mean | sd | mean | sd |
| *Eranthis hyemalis* | 57.5 | 21.6 | 59.3 | 23.9 |
| *Galanthus nivalis* | 58.0 | 18.5 | 70.6 | 18.3 |
| *Leucojum vernum* | 67.9 | 15.8 | NA | NA |
| *Anemone hepatica* | 77.1 | 17.4 | 88.8 | 13.7 |
| *Tussilago farfara* | 79.9 | 17.1 | 94.1 | 14.8 |
| *Viola odorata* | 87.7 | 14.8 | NA | NA |
| *Anemone nemorosa* | 94.3 | 15.2 | 100.6 | 11.2 |
| *Primula veris* | 119.7 | 9.3 | 120.3 | 10.3 |
| *Caltha palustris* | 120.4 | 10.0 | 118.4 | 8.8 |
| *Oxalis acetosella* | 120.9 | 10.5 | 114.9 | 6.2 |
| *Fragaria vesca* | 123.0 | 16.5 | 139.3 | 8.4 |
| *Convallaria majalis* | 130.0 | 11.2 | 144.6 | 8.8 |
| *Vaccinium myrtillus* | 130.5 | 11.0 | 136.1 | 9.4 |
| *Rubus idaeus* | 147.9 | 14.4 | 161.8 | 9.5 |
| *Dactylis glomerata* | 149.9 | 15.7 | 161.0 | 9.5 |
| *Trifolium pratense* | 151.0 | 15.9 | 162.9 | 8.3 |
| *Vaccinium vitis-idaea* | 151.0 | 8.9 | 151.8 | 9.9 |
| *Leucanthemum vulgare* | 160.0 | 9.1 | 160.8 | 8.3 |
| *Epilobium angustifolium* | 179.5 | 8.5 | 179.6 | 8.2 |
| *Artemisia vulgaris* | 200.6 | 18.5 | 206.0 | 9.0 |
| *Cyclamen purpurascens* | 210.4 | 21.3 | NA | NA |
| *Calluna vulgaris* | 222.8 | 15.6 | 215.5 | 13.5 |
| *Colchicum autumnale* | 246.1 | 16.0 | NA | NA |

**Table S1.** Mean and standard deviation of the day of the year (DOY) from the phenophases “Beginning of flowering: first flowers open” and “Beginning of flowering: 10% of flowers open.” NAs means there is no data of the phenophase for that species. The order of the species is based on the DOY mean of the first flower open.

Sampling

**Results of the T.G slope sampling:**

| Dataset | JS distance S.G - T.G | mean of JS distance S.G - Sampling T.G | p-value |
| --- | --- | --- | --- |
| S.G & T.G | 0.328 | 0.338 | **< 0.001** |
| S.G & T.G 1950-1985 | 0.342 | 0.356 | **< 0.001** |
| S.G & T.G 1986-2022 | 0.263 | 0.274 | **< 0.001** |

**Table S2.** Overview of the Jensen-Shannon (JS) distances when using spatial gradient (S.G) and temporal gradient (T.G) slopes, and when using the T.G slope samples and the S.G slopes. In total, 1000 slope samples from T.G were created with the same N size as in S.G slopes. The mean JS distances between the slope samples and S.G slopes are shown.

| Specie | JS distance S.G - T.G | mean of JS distance S.G - Sampling T.G | p-value |
| --- | --- | --- | --- |
| *Anemone hepatica* | 0.390 | 0.372 | **< 0.001** |
| *Anemone nemorosa* | 0.071 | 0.091 | **< 0.001** |
| *Artemisia vulgaris* | 0.153 | 0.184 | **< 0.001** |
| *Colchicum autumnale* | 0.129 | 0.179 | **< 0.001** |
| *Convallaria majalis* | 0.178 | 0.204 | **< 0.001** |
| *Caltha palustris* | 0.237 | 0.175 | **< 0.001** |
| *Cyclamen purpurascens* | 0.178 | 0.330 | **< 0.001** |
| *Calluna vulgaris* | 0.361 | 0.121 | **<0.01** |
| *Dactylis glomerata* | 0.389 | 0.357 | **< 0.001** |
| *Epilobium angustifolium* | 0.154 | 0.214 | **< 0.001** |
| *Eranthis hyemalis* | 0.209 | 0.273 | **< 0.001** |
| *Fragaria vesca* | 0.255 | 0.251 | **0.032** |
| *Galanthus nivalis* | 0.112 | 0.106 | **< 0.001** |
| *Leucojum vernum* | 0.120 | 0.356 | **< 0.001** |
| *Leucanthemum vulgare* | 0.396 | 0.140 | **< 0.001** |
| *Oxalis acetosella* | 0.256 | 0.227 | **< 0.001** |
| *Primula veris* | 0.241 | 0.234 | **< 0.001** |
| *Rubus idaeus* | 0.101 | 0.121 | **< 0.001** |
| *Tussilago farfara* | 0.334 | 0.113 | **< 0.001** |
| *Trifolium pratense* | 0.103 | 0.325 | **< 0.001** |
| *Vaccinium myrtillus* | 0.097 | 0.113 | **< 0.001** |
| *Viola odorata* | 0.165 | 0.396 | **< 0.001** |
| *Vaccinium vitis-idaea* | 0.433 | 0.178 | **< 0.001** |
| ALL SPECIES | 0.215 | 0.096 | **< 0.001** |

**Table S3.** Overview of the Jensen-Shannon (JS) distances per species when using spatial gradient (S.G) and temporal gradient (T.G) slopes, and when using the T.G slope samples and the S.G slopes. In total, 1000 slope samples from T.G were created with the same N size as in S.G slopes. The mean JS distances between the slope samples and S.G slopes are shown.

| Period:1950-1985 | | | |
| --- | --- | --- | --- |
| specie | JS distance S.G - T.G | mean JS distance S.G - Sampling T.G | p-value |
| *Anemone hepatica* | 0.455 | 0.404 | **< 0.001** |
| *Anemone nemorosa* | 0.121 | 0.120 | **0.031** |
| *Colchicum autumnale* | 0.128 | 0.084 | **< 0.001** |
| *Convallaria majalis* | 0.315 | 0.301 | **< 0.001** |
| *Calluna vulgaris* | 0.142 | 0.086 | **< 0.001** |
| *Dactylis glomerata* | 0.357 | 0.325 | **< 0.001** |
| *Galanthus nivalis* | 0.154 | 0.107 | **< 0.001** |
| *Rubus idaeus* | 0.055 | 0.100 | **< 0.001** |
| *Tussilago farfara* | 0.173 | 0.147 | **< 0.001** |
| ALL SPECIES | 0.193 | 0.126 | **< 0.001** |
| Period: 1986-2022 | | | |
| *Anemone hepatica* | 0.278 | 0.268 | **< 0.001** |
| *Anemone nemorosa* | 0.203 | 0.206 | **0.016** |
| *Colchicum autumnale* | 0.214 | 0.190 | **< 0.001** |
| *Convallaria majalis* | 0.304 | 0.304 | 0.166 |
| *Calluna vulgaris* | 0.128 | 0.110 | **< 0.001** |
| *Dactylis glomerata* | 0.488 | 0.445 | **< 0.001** |
| *Galanthus nivalis* | 0.089 | 0.089 | **0.028** |
| *Rubus idaeus* | 0.274 | 0.277 | 0.077 |
| *Tussilago farfara* | 0.135 | 0.135 | 0.925 |
| ALL | 0.078 | 0.110 | **< 0.001** |

**Table S4.** Overview of the Jensen-Shannon (JS) distances for the 1950-1985 and 1986-2022 periods when using spatial gradient (S.G) and temporal gradient (T.G) slopes, and when using the T.G slope samples and the S.G slopes. In total, 1000 slope samples from T.G were created with the same N size as in S.G slopes. The mean JS distances between the slope samples and S.G slopes are shown.

Species-specific responses

**Fig. S1** Phenological response to temperature per species. Density plots of the model slopes per species for the spatial gradient (S.G) data in red and for the temporal gradient (T.G) data in blue. Each species has their own response pattern; it could be very similar across both gradients, like in *Anemone nemorosa* (*A. nemorosa*) and *Tussilago farfara* (*T. farfara*), or divergent like in *Colchicum autumnale* (*C. autumnale*) and *Dactylis glomerata* (*D. glomerata*), or different in magnitude like in *Trifolium pratense* (*T. pratense*).

| **Dataset** | **Species** | **Mean Slope** | **N° sites** | **N° years** | **N° obs** |
| --- | --- | --- | --- | --- | --- |
| S.G | Anemone hepatica | 9.64 | 535 | 68 | 5611 |
|  | Anemone nemorosa | -6.88 | 6169 | 73 | 131797 |
|  | Artemisia vulgaris | 32.30 | 2419 | 32 | 31190 |
|  | Calluna vulgaris | 5.66 | 5429 | 72 | 99996 |
|  | Caltha palustris | -1.63 | 65 | 10 | 261 |
|  | Colchicum autumnale | -16.28 | 4671 | 73 | 62788 |
|  | Convallaria majalis | -6.30 | 526 | 71 | 5989 |
|  | Cyclamen purpurascens | -26.29 | 271 | 50 | 2382 |
|  | Dactylis glomerata | -8.34 | 744 | 73 | 7449 |
|  | Epilobium angustifolium | -20.11 | 61 | 10 | 197 |
|  | Eranthis hyemalis | -4.66 | 23 | 9 | 68 |
|  | Fragaria vesca | -4.82 | 297 | 49 | 2332 |
|  | Galanthus nivalis | -14.60 | 6662 | 73 | 163376 |
|  | Leucanthemum vulgare | -16.73 | 71 | 10 | 240 |
|  | Leucojum vernum | 9.29 | 448 | 60 | 4808 |
|  | Oxalis acetosella | -5.36 | 15 | 8 | 43 |
|  | Primula veris | -2.47 | 92 | 18 | 399 |
|  | Rubus idaeus | -10.50 | 4682 | 66 | 56133 |
|  | Trifolium pratense | -11.82 | 432 | 54 | 4817 |
|  | Tussilago farfara | -8.27 | 6665 | 73 | 140738 |
|  | Vaccinium myrtillus | -5.72 | 145 | 47 | 811 |
|  | Vaccinium vitis-idaea | -11.81 | 94 | 36 | 436 |
|  | Viola odorata | 5.07 | 407 | 50 | 5624 |
| T.G | Anemone hepatica | 1.19 | 422 | 70 | 5406 |
|  | Anemone nemorosa | -7.61 | 5601 | 73 | 130726 |
|  | Artemisia vulgaris | 23.32 | 1980 | 32 | 30416 |
|  | Calluna vulgaris | -2.50 | 4624 | 72 | 98530 |
|  | Caltha palustris | -2.72 | 31 | 11 | 196 |
|  | Colchicum autumnale | -20.89 | 3760 | 73 | 61128 |
|  | Convallaria majalis | -6.14 | 424 | 71 | 5791 |
|  | Cyclamen purpurascens | -15.63 | 172 | 50 | 2214 |
|  | Dactylis glomerata | -6.07 | 540 | 73 | 7102 |
|  | Epilobium angustifolium | -20.23 | 22 | 11 | 134 |
|  | Eranthis hyemalis | -23.36 | 7 | 10 | 43 |
|  | Fragaria vesca | -5.55 | 194 | 49 | 2151 |
|  | Galanthus nivalis | -13.40 | 6416 | 73 | 162879 |
|  | Leucanthemum vulgare | -15.77 | 25 | 10 | 158 |
|  | Leucojum vernum | 3.32 | 344 | 60 | 4620 |
|  | Oxalis acetosella | -6.16 | 5 | 10 | 33 |
|  | Primula veris | -4.08 | 47 | 18 | 317 |
|  | Rubus idaeus | -7.82 | 3736 | 70 | 54407 |
|  | Trifolium pratense | -9.84 | 354 | 57 | 4677 |
|  | Tussilago farfara | -8.61 | 6121 | 73 | 139674 |
|  | Vaccinium myrtillus | -6.05 | 85 | 50 | 714 |
|  | Vaccinium vitis-idaea | -12.87 | 48 | 46 | 377 |
|  | Viola odorata | -1.46 | 362 | 50 | 5541 |

**Table S5.** Overview of the data per species in the spatial (S.G) and temporal (T.G) gradient. Mean of the slopes from the linear mixed effect model, number of sites (N° Sites), years (N° Years), and observations (N° Obs). The observation number is the amount of flowering phenology records per species, site, and year.

**Table S6.** Results of the linear mixed-effects models comparing the response of the flowering onset to temperature across the spatial and temporal gradient. Variance of the random effects and estimates of the fixed effects from the linear mixed-effects models when the species are included in the random effect. SD: Standard deviation. SE: standard error. Estimates of T_Annual_ are in days/SD of °C. Models formula: lmer (DOY_Flowering_ ~ T_Annual_ + (0 + T_Annual_ | species_year)) for the spatial gradient, lmer (DOY_Flowering_ ~ T_Annual_ + (0 + T_Annual_ | species_site)) for the temporal gradient.

| **Spatial Gradient** | | | | | **Temporal Gradient** | | | | |
| --- | --- | --- | --- | --- | --- | --- | --- | --- | --- |
| *Random Effects* | *Obs* | *Variance* | *SD* | *p-value* | *Random Effects* | *Obs* | *Variance* | *SD* | *p-value* |
| sp_years | 1085 | 843.81 | 29.05 | **<0.001** | sp_sites | 717234 | 2002 | 44.75 | **<0.001** |
| Residual |  | 3213.80 | 56.69 |  | Residual |  | 2936 | 54.19 |  |
| *Fixed Effects* |  | *Estimates* | *SE* | *p-value* | *Fixed Effects* |  | *Estimates* | *SE* | *p-value* |
| Intercept |  | 123.31 | 0.08 | **<0.001** | Intercept |  | 123.38 | 0.08 | **<0.001** |
| T_Annual_ |  | -6.24 | 0.91 | **<0.001** | T_Annual_ |  | -9.19 | 0.26 | **<0.001** |
| *Marginal R^2^* | 0.010 | *Conditional R^2^* | | 0.215 | *Marginal R^2^* | 0.017 | *Conditional R^2^* | | 0.415 |

**Fig. S2** Density plot and boxplot of the spatial (red) and temporal (blue) gradient slopes. When the species are included in the random effect, the phenological response has high variation in both gradients. The Jensen-Shannon (JS) distance between both distributions is 0.215, meaning they are 78.5% similar.

Effects of climate change on space-time equivalence

| **Period: 1950-1985** | | | | | | | | | |
| --- | --- | --- | --- | --- | --- | --- | --- | --- | --- |
| **Spatial Gradient** | | | | | **Temporal Gradient** | | | | |
| *Random Effects* | *Obs* | *Variance* | *SD* | *p-value* | *Random Effects* | *Obs* | *Variance* | *SD* | *p-value* |
| sp_years | 322 | 885 | 29.75 | **0.003** | sp_sites | 27843 | 1995 | 44.67 | **<0.001** |
| Residual |  | 3713 | 60.94 |  | Residual |  | 3051 | 55.24 |  |
| *Fixed Effects* |  | *Estimates* | *SE* | *p-value* | *Fixed Effects* |  | *Estimates* | *SE* | *p-value* |
| Intercept |  | 129.69 | 0.15 | **<0.001** | Intercept |  | 130.32 | 0.11 | **<0.001** |
| T_Annual_ |  | -4.97 | 0.87 | **<0.001** | T_Annual_ |  | -7.94 | 0.29 | **<0.001** |
| *Marginal R^2^* | 0.005 | *Conditional R^2^* | | 0.197 | *Marginal R^2^* | 0.012 | *Conditional R^2^* | | 0.403 |
| **Period: 1986-2022** | | | | | | | | | |
| *Random Effects* | *Obs* | *Variance* | *SD* | *p-value* | *Random Effects* | *Obs* | *Variance* | *SD* | *p-value* |
| sp_years | 320 | 873 | 29.55 | **<0.001** | sp_sites | 15184 | 1346 | 37 | **<0.001** |
| Residual |  | 2760 | 52.54 |  | Residual |  | 2528 | 50.28 |  |
| *Fixed Effects* |  | *Estimates* | *SE* | *p-value* | *Fixed Effects* |  | *Estimates* | *SE* | *p-value* |
| Intercept |  | 102.44 | 0.13 | **<0.001** | Intercept |  | 103.47 | 0.13 | **<0.001** |
| T_Annual_ |  | -10.06 | 1.68 | **<0.001** | T_Annual_ |  | -10.87 | 0.33 | **<0.001** |
| *Marginal R^2^* | 0.027 | *Conditional R^2^* | | 0.261 | *Marginal R^2^* | 0.030 | *Conditional R^2^* | | 0.367 |

**Table S7.** Results of the linear mixed-effects models comparing the response of the flowering onset to temperature across the spatial and temporal gradient in the 1950-1985 and 1986-2022 periods. Variance of the random effects and estimates of the fixed effects from the linear mixed-effects models in the 1950-1985 and 1986-2022 periods across both gradients. SD: Standard deviation. SE: standard error. Estimates of T_Annual_ are in days/SD of °C. Models formula: lmer (DOY_Flowering_ ~ T_Annual_ + (0 + T_Annual_ | year)) for the spatial gradient, lmer (DOY_Flowering_ ~ T_Annual_ + (0 + T_Annual_ | site)) for the temporal gradient.

| **Period: 1950-1985** | | | | | | | | | |
| --- | --- | --- | --- | --- | --- | --- | --- | --- | --- |
| **Spatial Gradient** | | | | | **Temporal Gradient** | | | | |
| *Random Effects* | *Obs* | *Variance* | *SD* | *p-value* | *Random Effects* | *Obs* | *Variance* | *SD* | *p-value* |
| Years | 36 | 7.31 | 2.703 | **<0.001** | Sites | 5983 | 91.86 | 9.58 | **<0.001** |
| Residual |  | 4973 | 70.52 |  | Residual |  | 4884.63 | 69.89 |  |
| *Fixed Effects* |  | *Estimates* | *SE* | *p-value* | *Fixed Effects* |  | *Estimates* | *SE* | *p-value* |
| Intercept |  | 129.59 | 0.12 | **<0.001** | Intercept |  | 129.51 | 0.13 | **<0.001** |
| T_Annual_ |  | -7.64 | 0.46 | **<0.001** | T_Annual_ |  | -9.16 | 0.18 | **<0.001** |
| *Marginal R^2^* | 0.012 | *Conditional R^2^* | | 0.013 | *Marginal R^2^* | 0.017 | *Conditional R^2^* | | 0.035 |
| **Period: 1986-2022** | | | | | | | | | |
| *Random Effects* | *Obs* | *Variance* | *SD* | *p-value* | *Random Effects* | *Obs* | *Variance* | *SD* | *p-value* |
| Years | 37 | 27.32 | 5.227 | **<0.001** | Sites | 4019 | 100.21 | 10.01 | **<0.001** |
| Residual |  | 3792 | 61.58 |  | Residual |  | 3675.05 | 60.62 |  |
| *Fixed Effects* |  | *Estimates* | *SE* | *p-value* | *Fixed Effects* |  | *Estimates* | *SE* | *p-value* |
| Intercept |  | 102.59 | 0.15 | **<0.001** | Intercept |  | 102.86 | 0.15 | **<0.001** |
| T_Annual_ |  | -8.27 | 0.87 | **<0.001** | T_Annual_ |  | -10.78 | 0.23 | **<0.001** |
| *Marginal R^2^* | 0.018 | *Conditional R^2^* | | 0.025 | *Marginal R^2^* | 0.017 | *Conditional R^2^* | | 0.035 |

**Table S8.** Results of the linear mixed-effects models comparing the response of the flowering onset to temperature across the spatial and temporal gradient in the 1950-1985 and 1986-2022 periods. Variance of the random effects and estimates of the fixed effects for both gradients are shown. SD: Standard deviation. SE: standard error. Estimates of T_Annual_ are in days/SD of °C. Models formula: lmer (DOY_Flowering_ ~ T_Annual_ + (0 + T_Annual_ | year)) for the spatial gradient, lmer (DOY_Flowering_ ~ T_Annual_ + (0 + T_Annual_ | site)) for the temporal gradient.

**Fig. S3** Models slope distribution per species in the spatial gradient (S.G) in red, and in the temporal gradient (T.G) in blue for the periods of **a.** 1950 to 1986 and **b.** 1986 to 2022.
